# The physiological cost of coping: a systematic review and meta-analysis on discrimination and heart rate variability

**DOI:** 10.1093/abm/kaag058

**Published:** 2026-09-24

**Authors:** Giuseppe Salvo, Daniele Bomarsi, Fiorenzo Laghi, Gianluca Esposito, Julian F Thayer, Roberto Baiocco, Cristina Ottaviani

**Affiliations:** Department of Developmental and Social Psychology, Sapienza University of Rome, Rome 00185, Italy; Department of Psychology, Sapienza University of Rome, Rome 00185, Italy; Department of Developmental and Social Psychology, Sapienza University of Rome, Rome 00185, Italy; Department of Psychology and Cognitive Science, University of Trento, Rovereto 38068, Italy; Department of Psychology, University of California, Irvine, Irvine, CA 92697, United States; Department of Developmental and Social Psychology, Sapienza University of Rome, Rome 00185, Italy; Department of Psychology, Sapienza University of Rome, Rome 00185, Italy; LIFE Institute for Research and Health Care, Santa Lucia IRCCS, Rome 00179, Italy

**Keywords:** cardiovascular conundrum, health disparities, marginalization, meta-analysis, minority stress, parasympathetic regulation

## Abstract

**Background:**

Heart rate variability (HRV) reflects parasympathetic regulation and is typically associated with better health outcomes. However, minoritized populations, particularly African Americans, show a paradoxical pattern of higher resting HRV despite greater cardiovascular risk, a phenomenon known as the *Cardiovascular Conundrum*. Discrimination has been proposed as a key mechanism, but evidence remains fragmented.

**Methods:**

The present meta-analysis quantified HRV in relation to discrimination across 3 domains: (1) resting HRV differences between minoritized and majority groups, (2) HRV reactivity to discrimination-related tasks, and (3) the association between perceived discrimination and resting HRV. A systematic search yielded 40 articles comprising 52 independent studies.

**Results:**

Minoritized groups showed higher resting HRV than majority groups (*g* = .22, *P* < .001), as well as acute HRV reductions during discrimination-related tasks (*g *= −.17, *P* = .04), and lower resting HRV associated with greater perceived discrimination (*g *= −.19, *P* = .006). Evidence of publication bias emerged only for the latter meta-analysis. No significant moderators emerged to explain the substantial heterogeneity, with the exception of the type of HRV measure used.

**Conclusions:**

These findings are consistent with the hypothesis that discrimination may become biologically embedded through acute vagal withdrawal during discriminatory events, a cumulative reduction in HRV associated with perceived discrimination, and a paradoxical elevation of resting HRV indicative of chronic compensatory regulation.

## Introduction

For many individuals who are *minoritized*—whether on the basis of race, sexual identity, gender, or other socially marked identities—discrimination, overt or subtle, is an embodied experience that shapes how one inhabits space, anticipates interaction, and regulates emotions from moment to moment. It has been described as a lived experience of friction, disorientation, and alienation, characterized by external judgment and internal vigilance.^1^^,^^2^ The term *minoritized* is preferred over “minority” to shift the focus from a purely numerical or quantitative underrepresentation to the systemic power asymmetries and social practices that structurally disadvantage specific populations.^3^ Accordingly, this concept broadly encompasses both traditional minority groups and socially stigmatized populations, as both are marginalized by these power dynamics rather than any intrinsic characteristics.

Such experiences evoke chronic anger, shame, fear, and hypervigilance, all of which may require sustained emotion regulation. Importantly, this phenomenological dimension is not merely subjective: repeated vigilance and ongoing emotion regulation demands are closely linked to autonomic processes involved in stress adaptation, gradually modulating physiological functioning over time.

According to the Biopsychosocial Model of Racism, discrimination-related stress is associated with disruptions in autonomic functioning and with an increased risk of elevated blood pressure and cardiovascular disease (CVD).^4–6^ For historical reasons, the minoritized population that has received the most attention is that of African Americans (AAs), who are subjected to greater hypertension-related complications^5^^,^^7^ and a higher burden of CVD.^8^

In this specific minoritized group, studies have repeatedly identified a paradoxical autonomic profile: despite being at increased risk of cardiovascular morbidity and mortality, AAs consistently exhibit higher resting vagally mediated heart rate variability (HRV), an index of parasympathetic cardiac regulation that is typically considered cardioprotective. Further complicating this picture, higher resting HRV in AAs has also been associated with increased total peripheral resistance (TPR), despite the fact that parasympathetic regulation and vascular resistance are generally expected to show an inverse physiological relationship. This paradoxical pattern—referred to as the *Cardiovascular Conundrum*^9^^,^^10^—has traditionally been interpreted in terms of genetic influences.^11^ However, more recent findings challenge this interpretation by showing similar autonomic profiles in socially minoritized White individuals.^12^ The presence of the conundrum across racially distinct but socially marginalized groups suggests that environmental marginalization and chronic minority stress, rather than race-specific genetic factors alone, may contribute to this paradoxical physiological profile.

Emotion regulation strategies may help explain these paradoxical findings. Coping responses such as cognitive reappraisal, emotional suppression, and rumination have been systematically linked to variations in HRV,^13^ with evidence showing both increases and decreases in HRV depending on the specific strategy employed. In particular, perseverative cognition—defined as the sustained or repetitive activation of cognitive representations of feared events in the future or past stressful events^14^^,^^15^ has been consistently linked to lower HRV.^16^ By contrast, cognitive reappraisal and emotional suppression have been associated with increased HRV,^17^ although the magnitude and meaning of these effects appear to be culturally mediated.^18^ Within this framework, anger inhibition has been described as a context-dependent form of emotional suppression frequently employed by AAs to navigate discriminatory environments.^19^^,^^20^ Notably, although anger inhibition is associated with higher resting HRV, it also predicts increased vascular resistance and delayed TPR recovery,^20^ a pattern consistent with the so-called “vigilance response,” characterized by heightened attention to potential threat and previously linked to chronically elevated vascular tone and prolonged perseverative cognition.^14^ Importantly, increased TPR is strongly associated with long-term morbidity and mortality related to hypertension.^21^^,^^22^ The chronic regulatory demands imposed by repeated exposure to discrimination are therefore hypothesized to interact with these autonomic pathways over time.

Within this framework, elevated HRV in minoritized individuals is not necessarily interpreted as reflecting greater adaptive regulatory capacity. Rather, it may reflect ongoing, high-effort compensatory regulation under conditions of chronic vigilance and stress. Although the available evidence is largely correlational, repeated engagement of these regulatory processes has been hypothesized to contribute over time to the paradoxical phenotype of elevated HRV co-occurring with heightened vascular resistance.^10^^,^^20^^,^^23^

Although most research has focused on racial disparities, the Minority Stress Model^24^ provides a broader framework for examining the associations between discrimination and autonomic functioning across groups. Sexual minorities, for example, face both distal stressors (eg, overt discrimination, victimization) and proximal stressors (eg, vigilance, internalized stigma), which are hypothesized to cumulatively alter the autonomic nervous system.^25^ Accordingly, preliminary evidence suggests that lesbian, gay, and bisexual (LGB) individuals also exhibit elevated resting HRV alongside higher TPR compared to their heterosexual counterparts,^12^ indicating that the cardiovascular conundrum may generalize across minoritized populations and reflect a broader psychophysiological signature of minority stress.

## The present study

The present meta-analysis aimed to quantify the magnitude of these effects and examine potential moderators in order to better understand how experiences of marginalization may become ­biologically embedded.

To the best of our knowledge, only one previous systematic review has examined the impact of discrimination across a broad range of cardiovascular outcomes, including blood pressure, circulating and salivary biomarkers, and heart rate variability.^26^ However, that review included only a small number of studies assessing HRV (*k* = 6), reflecting the limited evidence available at the time. Moreover, whereas the previous review provided a primarily descriptive synthesis across heterogeneous cardiovascular outcomes, the present study offers a formal meta-analytic quantification of effect sizes across 3 distinct analytic frameworks (group differences in resting HRV, phasic HRV responses to discrimination-related stimuli, and associations between perceived discrimination and resting HRV). This approach allows for a more precise characterization of the magnitude, direction, and heterogeneity of effects, and provides a more detailed understanding of how discrimination is associated with parasympathetic regulation.

Research on HRV in minoritized populations has broadly examined 2 complementary dimensions of parasympathetic functioning: tonic HRV and phasic HRV responses. Tonic HRV refers to resting parasympathetic regulation and is generally interpreted as a relatively stable marker of regulatory functioning and stress adaptation.^27^ By contrast, phasic HRV reflects dynamic moment-to-moment changes in vagal regulation in response to acute stressors, including discriminatory experiences.^28^ This distinction is particularly relevant in the context of minority stress, as acute exposure to discrimination may trigger transient vagal withdrawal, whereas chronic exposure may shape longer-term patterns of autonomic regulation through sustained emotion regulation demands and vigilance processes.

To provide a comprehensive understanding of the phenomenon, the present meta-analysis integrates these dimensions through a 3-fold approach: comparing resting (tonic) HRV between minoritized and majority groups (meta-analysis 1); assessing phasic HRV responses to discrimination-related stimuli (meta-analysis 2); and testing associations between perceived discrimination and resting (tonic) HRV (meta-analysis 3). Through this integrative framework, this systematic review aims to clarify the pathways linking discrimination to autonomic regulation, advancing our understanding of how the lived experience of marginalization is reflected in both tonic and phasic HRV.

Among possible moderators, the potential role of age warrants investigation, as HRV is known to decline across the lifespan; however, this decrease appears to be more pronounced in EAs than in AAs.^9^^,^^29–31^ With regard to sex-related differences, women tend to show higher HRV than men, despite exhibiting a higher HR.^32^ In this regard, the “cardiovascular conundrum” may be more pronounced in women than in men, since AA women tend to have higher baseline blood pressure and vascular resistance measures compared to AA men, despite higher HRV.^33^ Across populations, elevated BMI is generally associated with reduced parasympathetic activity, reflected in lower HRV and higher resting HR compared with normal-weight individuals.^34^^,^^35^ Yet, the strength of this inverse association seems to vary by ethnicity. In EAs, the association between BMI and HRV appears stronger and more linear, whereas in AAs the decline in HRV with increasing BMI is less pronounced.^29^ Additionally, examining the potential moderating effects of the country in which the study was conducted, ethnicity, health status, and minority type (eg, ethnic vs. sexual minority status) is important, as sociocultural context, baseline health conditions, and the specific forms of stigma and discrimination experienced by different groups may shape parasympathetic responses to minority stress.

## Methods

### Identification and selection of studies

Two search strategies were employed to systematically identify empirical studies examining the relationship between HRV and discrimination. The first electronic search was conducted in the PsycArticles, PsycInfo, PubMed, and Web of Science databases up to February 16, 2024. An updated search using the same strategy was conducted on April 16, 2026. The search string combined terms related to HRV and discrimination: (“heart rate variability” OR HRV) AND (discrimination OR prejudice OR racism OR sexism OR minorities OR “minority group” OR “minority people” OR “minority individuals” OR minoritized OR minorized OR marginalized OR marginalised OR stigmatized OR stigmatised). Second, the reference lists of prior systematic reviews were manually screened to identify additional relevant studies.

The search was restricted to English language, peer-reviewed publications reporting on human samples; therefore, gray literature and unpublished studies were systematically excluded. Studies were eligible for inclusion if they met the following criteria:

1. employed a design permitting the calculation of one or more effect sizes (eg, randomized within-subjects designs, case-control designs, or correlational studies);
2. incorporated a comparator condition, such as minoritized versus majority groups or control condition versus discrimination-related tasks or the correlation between self-reported discrimination and tonic HRV levels;
3. assessed HRV outcomes using root mean square of successive differences (RMSSD), high-frequency HRV (HF-HRV), or respiratory sinus arrhythmia (RSA). Only vagally mediated HRV indices were included, because these measures are considered the most reliable indicators of parasympathetic cardiac regulation. Non-specific indices (eg, SDNN, LF/HF ratio) were excluded due to their mixed physiological interpretation and limited specificity. When multiple eligible HRV indices were reported within the same study, preference was given to RMSSD, followed by HF-HRV and then RSA, based on their wider use and comparability across studies.

**Figure 1:**
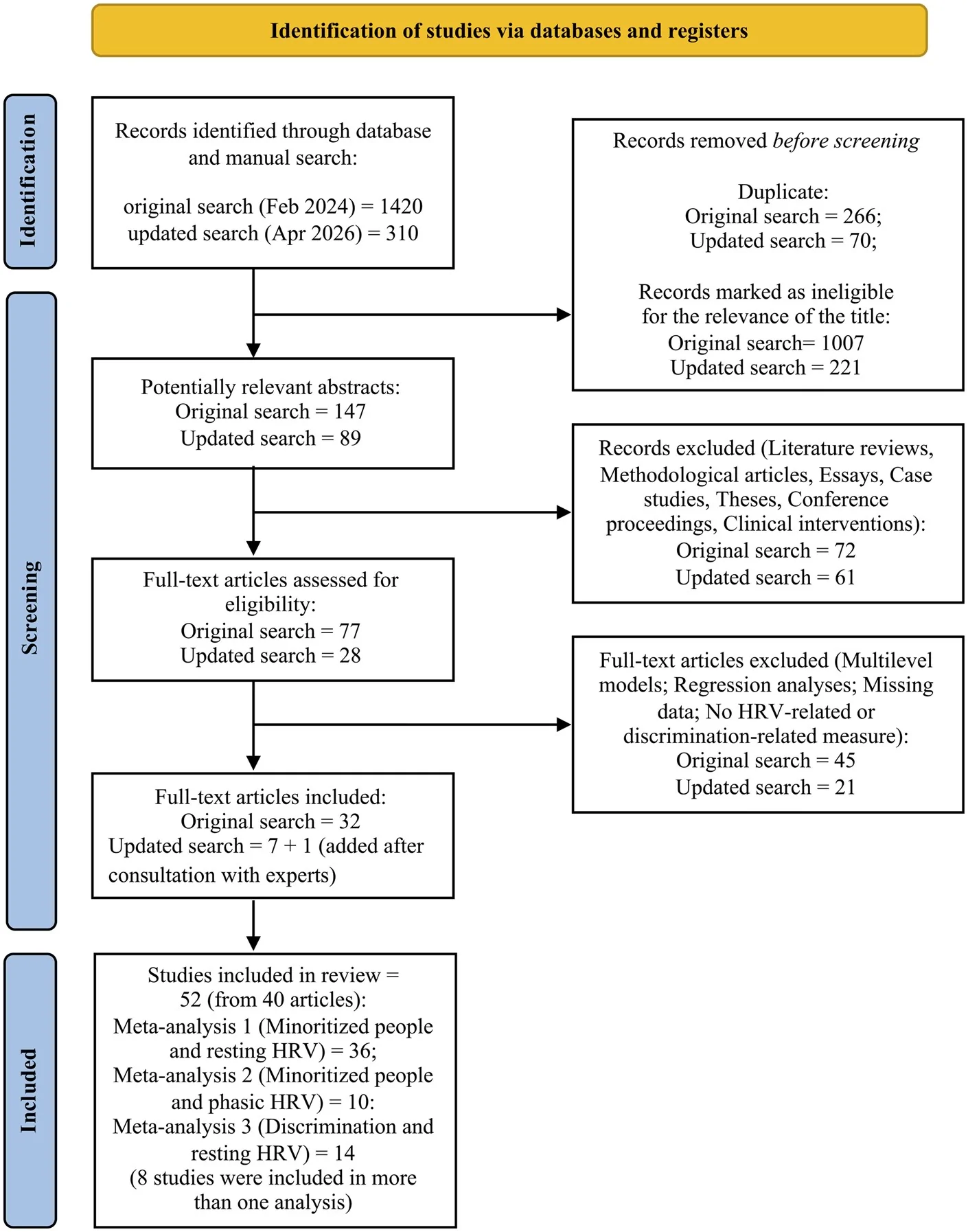
PRISMA flowchart describing search results and study selection.

As shown in Figure 1, the initial search yielded 1420 records. Following the removal of 266 duplicates, 1154 records were screened at the title and abstract level, resulting in 77 articles for full-text assessment and 32 articles selected. The updated search yielded 310 new records. Following the removal of 70 duplicates, 240 records were screened at the title and abstract level, resulting in 28 articles for full-text assessment, resulting in the inclusion of 7 additional articles. Through consultation with experts in the field, one additional eligible peer-reviewed article,^18^ which had not been captured by the initial electronic database search, was identified and included in the final sample. The present meta-analyses are based on data extracted from 40 articles comprising 52 independent studies that met inclusion criteria (Table 1). Among the 52 studies, 36 were included in meta-analysis 1 (M1), 10 in meta-analysis 2 (M2), and 14 in meta-analysis 3 (M3) (Figure 1 and Supplementary Material for the PRISMA flowchart).

**Table 1:** Studies included in the meta-analyses and conditions/comparisons used to derive effect sizes.

Authors	Year	M-A	*N*	Age	F (%)	Co.	Status	BMI	Type of Mnr	Exp. group	Contr. group	Des	Assessment	Exp. condition	Control condition	Perceived discrimin.	HRV measure	Hedges’ *g*
**Bell et al.^78^**	2019	M3	57	22.9	54.4	USA	Healthy	25.4	Ethnic	AA		C	At rest			Perceived racism scale^79^	HF-HRV	−0.09
**Brownlow et al.^80^**	2024	M3	238	20.9	67.3	USA	n.s.	27.8	Ethnic	AA		C	At rest			Race-related vigilance^81^	RMSSD	0.06
**Christian et al.^33^**	2020	M1	36		100	USA	Healthy		Ethnic	AA (19)	EA (17)	B	At rest				HF-HRV	0.76
**Cooper et al.^54^**	2014	M2	81	19.8	100	USA	Healthy	24.1	Ethnic	AA		W	At rest/Task	Racism recall task	Baseline		HRV	−0.66
**Dorr et al.^20^**	2007	M1/M2	50			USA	Healthy		Ethnic	AA (24)	EA (26)	B/W	At rest/Task	Racial related debate stressor	Baseline		HRV	0.55/−0.50
**Doucerain et al.^39^**	2022	M2/M3	111	30.5	54.05	CAN	n.s.	23.7	Ethnic	M-A-T		W/C	At rest/Task	Discrim. recall task	Baseline		RSA	−0.29/−0.03
**Earnest et al.^82^**	2008	M1	350	57.5	100	USA	OW, OB		Ethnic	AA (102)	Cau. (248)	B	At rest				RMSSD	0.53
**Fuller-Rowell et al.^83^**	2013	M1	1037	53.8	57.2	USA	HTN		Ethnic	BA (204)	WA (833)	B	At rest				RMSSD	0.50
**Goreis et al.^40^**	2022	M2/M3	72	24.4		AUT	Healthy		Ethnic	Turkish—High (35)	Turkish—Low (37)	B/C	At rest/Task	Ethnic discrimin. paradigm	Baseline		RMSSD	0.01/−0.35
**Gutin et al. (a)^84^**	2005	M1	171	16.2	100	USA	Healthy	23.3	Ethnic	AA		B	At rest				RMSSD	0.24
**Gutin et al. (b)^84^**	2005	M1	133	16.2	0	USA	Healthy	22.8	Ethnic	AA		B	At rest				RMSSD	0.35
**Heffernan et al.^52^**	2009	M1	39	23		USA	Healthy	26.9	Ethnic	AA (18)	EA (21)	B	At rest				RMSSD	2.70
**Hill & Hoggard^69^**	2018	M3	69	20	100	USA	Healthy		Ethnic	AA		C	At rest			Everyday Discrimin. Scale^40^	vmHRV	−0.10
**Hill, Hoggard et al.^29^**	2017	M3	99	19.9	58	USA	Healthy	27.1	Ethnic	AA		C	At rest			Perceived Ethnic Discrimin. Question. Community Version (PEDQ-CV^86^	HF-HRV	−0.12
**Hill, Watkins et al.^85^**	2017	M1	148	33.2	43.9	USA	Healthy	26	Ethnic	AA (74)	WA (74)	B	At rest				InHF-HRV	0.16
**Kemp et al.^53^**	2016	M1	8487	52.1	55.5	BRA	D, Depr, Dysl, H-CHD, HTN	27.4	Ethnic	BBr (2020)	WBr (6467)	B	At rest				HF-HRV	0.30
**Li et al. (a)^87^**	2009	M1	209	23.1	100	USA	Healthy		Ethnic	AA (125)	EA (84)	B	At rest				RMSSD	−0.07
**Li et al. (b)^87^**	2009	M1	190	23.1	0	USA	Healthy		Ethnic	AA (85)	EA (105)	B	At rest				RMSSD	0.18
**Liao et al. (a)^88^**	1995	M1	573	50*	100	USA	ATS, D, HTN		Ethnic	BA (150)	WA (423)	B	At rest				HF Power	0.10
**Liao et al. (b)^88^**	1995	M1	448	50*	0	USA	ATS, D, HTN		Ethnic	BA (97)	WA (351)	B	At rest				HF Power	−0.19
**Liao et al. (c)^88^**	1995	M1	473	60*	100	USA	ATS, D, HTN		Ethnic	BA (114)	WA (359)	B	At rest				HF Power	0.16
**Liao et al. (d)^88^**	1995	M1	490	60*	0	USA	ATS, D, HTN		Ethnic	BA (79)	WA (411)	B	At rest				HF Power	−0.04
**Lin et al.^36^**	2024	M1/M2	38	19.6	100	USA	n.s.		Ethnic	AA (15)	WA (23)	W/B	At rest/Task	Discrimin. recall task	Baseline		HF-HRV	−0.36/0.47
**Pourmand et al.^89^**	2024	M1	429	19.4	43.12	USA	Healthy	24.7	Ethnic	AA (110)	EA (319)	B	At rest				HRV	0.19
**Rhudy et al.^38^**	2025	M1/M3	252	29.9	54	USA	Healthy	24.9	Ethnic	Native Americans (152)	NHW (130)	B/C	At rest			Everyday Discrimination Scale^40^	RMSSD	−0.07/0.01
**Rosati et al. (1)^12^**	2021	M1	58	19.8		USA	n.s.	26.2	Ethnic	AA (30)	EA (28)	B	At rest				RMSSD	0.48
**Rosati et al. (2)^12^**	2021	M1	39	35.3		ITA	n.s.	25.3	Sexual	LGB (19)	Hetero (20)	B	At rest				RMSSD	0.65
**Salomon (a)^44^**	2005	M1	49	11.5*	100	USA	Healthy		Ethnic	AA (24)	EA (25)	B	At rest				RSA	0.64
**Salomon (b)^44^**	2005	M1	51	11.5*	0	USA	Healthy		Ethnic	AA (22)	EA (29)	B	At rest				RSA	0.18
**Salomon (c)^44^**	2005	M1	24	19*	100	USA	Healthy		Ethnic	AA (9)	EA (15)	B	At rest				RSA	0.23
**Salomon (d)^44^**	2005	M1	25	19*	0	USA	Healthy		Ethnic	AA (10)	EA (15)	B	At rest				RSA	0.04
**Sosoo et al.^90^**	2022	M2	101	23.7	55.4	USA	Healthy		Ethnic	AA		W	At rest/Task	Photos of police violence against Blacks	Baseline		RSA	−0.08
**Thayer & Koenig^23^**	2019	M1	182	44.5	63.2	USA	n.s.	29.2	Ethnic	AA (102)	EA (80)	B	At rest				HF-HRVlog	−0.01
**Thayer, Watanabe et al.^91^**	2025	M1	255	23.1	55.25	USA	Healthy	28.4	Ethnic	AA (152)	EA (103)	B	At rest				HF-HRV	0.20
**Utsey & Hook (a)^76^**	2007	M3	116		100	USA	n.s.		Ethnic	AA		C	At rest			Index of Race-Related Stress—Brief Version (IRRS-B^92^)	RMSSD	−0.15
**Utsey & Hook (b)^76^**	2007	M3	82		0	USA	n.s.		Ethnic	AA		C	At rest			Index of Race-Related Stress—Brief Version (IRRS-B^92^)	RMSSD	−0.03
**Volpe et al.^41^**	2019	M2/M3	119	19.4	76.5	USA	n.s.	25.8	Ethnic	AA		W	At rest/Task	Racial discrimin. vignette	Baseline	Racism and Life Experiences Scale—Daily Life Experiences subscale (RaLES; Harrell, 1997)	RSA	−0.29/−0.08
**Volpert-Esmond et al.^55^**	2024	M2	83	20.9*	80.41	USA	Healthy		Ethnic	Latino/Hispanics		W	At rest/Task	Discrimination Speaking Task	Baseline	Past-year Racial/Ethnic Discrimination	RSA	0.03
**Wagner et al.^93^**	2015	M1	32	55.1	100	USA	D		Ethnic	AA (16)	WA (16)	B	At rest				HF-HRV	0.26
**Wang et al. (a)^31^**	2005	M1	196	15.5	100	USA	Healthy	23.5	Ethnic	AA (74)	EA (122)	B	At rest				RMSSD	0.36
**Wang et al. (b)^31^**	2005	M1	189	16	0	USA	Healthy	23	Ethnic	AA (77)	EA (112)	B	At rest				RMSSD	0.45
**Wang et al. (a)^94^**	2009	M1	399	18.2	100	USA	Healthy	24.9	Ethnic	AA (182)	EA (217)	B	At rest				RMSSD	0.16
**Wang et al. (b)^94^**	2009	M1	336	17.4	0	USA	Healthy	23.6	Ethnic	AA (126)	EA (210)	B	At rest				RMSSD	0.20
**Watanabe, Pourmand et al.^18^**	2023	M1	374	19.4	52.67	USA	Healthy	23.6	Ethnic	Asian American (63)	EA (311)	B	At rest				lnHF-HRV	−0.15
**Watanabe, Tyra et al.^95^**	2026	M1	636	19.8	60.64	USA	Healthy		Ethnic	AA (82)	EA (554)	B	At rest				lnHF-HRV	0.47
**Weissman et al.^45^**	2019	M3	229	17.2	48.03	USA	n.s.		ethnic	Mex-origin		C	At rest			Discrimin. frequency	RSA	
**Wesarg-Menzel et al.^56^**	2025	M3	67	28*	16.42	Germany	Healthy		Ethnic/Migrant	Arabic speaking refugees		C	At rest			Perceived Ethnic Discrimination Questionnaire (PEDQ; Contrada et al. 2001)	logRMSSD	−0.28
**Williams, Joseph et al.^63^**	2019	M2	43	20	55.8	USA	Healthy		Ethnic	AA		W	At rest/Task	Stereotype threat manipulation	Baseline		vmHRV	−0.360
**Williams, Pandya et al.^37^**	2019	M1/M3	45	19.8		USA	Healthy	25.2	Ethnic	AA		C	At rest			Perceived Ethnic Discrimin. Question.^86^	HF-HRV	0.17/−0.36
**Williams, Pourmand et al.^42^**	2025	M2/M3	108	20.5	58.23	USA	Healthy		Ethnic	Latin (85)	EA (23)	W/C	At rest/Task	Social evaluative speech task	Baseline	Experience of Discrimination Scale (EOD; Krieger et al., 2005)	RMSSD	0.01/−0.04
**Williams, Wiley et al.^96^**	2024	M1	130	19.7	41.54	USA	Healthy	25.5	Ethnic	AA (57)	EA (73)	B	At rest				RMSSD	0.45
**Zion et al.^52^**	2003	M1	61	22.7	0	USA	Healthy	24.4	Ethnic	AA (32)	Non-AA (29)	B	At rest				HF-HRV	−0.72

The numbers 1 or 2 following author names indicate an independent study from that article; the letters a, b, c, or d following author names indicate an independent sample with a non-overlapping stratification (eg, by gender or age) and an independent effect size.

Abbreviations: AA, African Americans; ATS, Atherosclerosis; AUT, Austria; B, between-subject; BA, Black American; BBr, Black Brazilian; BMI (tot.), mean of the total sample’s body mass index; BMI (mnr), mean of the minoritized group’s body mass index; BRA, Brazil; C, Correlational; CAN, Canada; Cau., Caucasian; Co., country where the study was conducted; D, diabetes; Depr., antidepressant use; Des., study design; DV, dependent variable; Dysl., dyslipidemia; *, estimated; H-CHD, hard coronary disease; Hetero, heterosexual; HF-HRV, high frequency heart rate variability; HTN, hypertension; ITA, Italy; LGB, Lesbian Gay bisexual; M1, meta-analysis 1; M2, meta-analysis 2; M3, meta-analysis 3; M1/M2, this abbreviation is used for those studies included in both meta-analyses M1 and M2; M1/M3, this abbreviation is used for those studies included in both meta-analyses M1 and M3; M2/M3, this abbreviation is used for those studies included in both meta-analyses M2 and M3; Mnr, minority; M-A, meta-analysis, which indicates the meta-analysis (meta-analysis 1, 2, or 3) where the article is included; M-A-T, born in Morocco, Algeria, or Tunisia; Mex, Mexican; N, sample size; Non-AA, Non-African American; n.s., not specified; OB, obesity; OW, overweight; RMSSD, root mean square of successive differences; RSA, respiratory sinus arrhythmia; USA, United States of America; vmHRV, vagally mediated heart rate variability; W, within-subject; WA, White American; WBr, White Brazilian.

Several studies contributed data to more than one analysis. Specifically, 2 studies^18^^,^^20^ assessed both resting HRV differences between groups and HRV reactivity to a discrimination-related task, contributing to M1 and M2. Similarly, 2 studies^37^^,^^38^ reported both resting HRV differences and the correlation between HRV and perceived discrimination, contributing to M1 and M3. Finally, 4 studies^39–42^ provided data on both task-related HRV changes and associations between resting HRV and perceived discrimination and were therefore included in M2 and M3.

### Coding

A standardized data coding form was developed to extract the following information from each study: (1) authors and year of publication; (2) study design; (3) sample characteristics (eg, age, sex assigned at birth, body mass index (BMI), sample size, subgroup composition, ethnic background, social status, minority type); (4) type of discrimination-related stimulus; (5) measures of perceived discrimination; (6) HRV measure; (7) outcomes of interest; and (8) a concise summary of findings. Each independent sample contributed only one effect size per meta-analysis.^43^ For this reason, in the case of a longitudinal study, only the first assessment with adequate data was used, with 2 exceptions: in Salomon,^44^ data from the second assessment were used because they provided a larger data set and more complete information (eg, sample size, age); and in Weissman et al.,^45^ we extracted the correlation between RSA at age 17 and perceived discrimination at age 16 (final wave of data collection) in order to minimize the temporal gap between the 2 measures.

All studies included in the meta-analysis examining differences in resting (tonic) HRV between minoritized and majority groups (M1) employed a between-subjects design. In all cases, minoritized participants were coded as the experimental group and majority participants as the control group. Most studies focused on ethnic minority populations; the only exception was Rosati et al. (Study 2),^12^ which investigated differences in resting HRV between LGB individuals (minoritized group) and heterosexuals (majority group).

All studies included in the meta-analysis examining changes in phasic HRV during discrimination-related tasks (M2) employed within-subjects designs conducted with minoritized participants. When studies had more than one condition (eg, baseline, discrimination-related task, recovery, neutral task, etc.), the HRV measurement during the first discrimination-related task was considered as the experimental condition, while the most neutral condition—resting or baseline HRV—was selected as the control condition.

Studies included in the meta-analysis assessing the association between perceived discrimination and resting HRV (M3) were predominantly correlational in design. The only exception was Goreis et al.,^40^ which compared 2 groups differentiated by levels of ethnic discrimination, as measured by the Everyday Discrimination Scale.^46^ Participants reporting chronic discrimination were classified as the high-discrimination group, which we treated as the experimental condition, while those reporting infrequent discrimination constituted the low-discrimination group, treated as the control condition.

Study selection and data extraction were conducted independently by 2 trained coders (G.S. and D.B.). To assess coding reliability, inter-rater agreement was calculated on a randomly selected subset comprising 30% of the included studies. The level of agreement was excellent, with a concordance rate of 98%. Any discrepancies identified during this process were resolved through discussion and consensus between the 2 coders.

### Data analysis

For each study (or subsample), effect sizes were computed using Hedges’ *g*. Consistent with Cohen’s criteria,^47^ values of 0.20, 0.50, and 0.80 were interpreted as small, medium, and large effects, respectively. In meta-analysis 1 (M1), *g* represented the difference in resting HRV between minoritized and majority groups. In meta-analysis 2 (M2), effect sizes reflected changes in HRV in response to discrimination-related stimuli (eg, discrimination recall tasks, racial discrimination vignettes, stereotype threat manipulations) relative to a control condition (eg, resting HRV). Positive values indicated HRV increases during the discrimination-related task, whereas negative values indicated HRV decreases. For correlational studies included in meta-analysis 3 (M3), Pearson’s *r* coefficients were converted to *g.*^48^ In Goreis et al.,^40^ positive values were assigned to effect sizes indicating higher HRV in the high-discrimination group compared with the low-discrimination group.

Effect sizes were calculated using means, standard deviations, mean differences, *P* values, and sample sizes when available. When only standard errors (SEs) were reported, standard deviations (SDs) were derived using the formula SD = SE × √*n.*^49^ If raw data were unavailable but alternative statistics (eg, *t* values) were reported, transformation formulas were applied to derive *g*.^48^ For results reported only as thresholds (eg, *P* < .05 or *P* < .001), Hedges’ *g* was estimated using conservative approximations of *P* values (.045 and .00095, respectively).

All analyses were conducted in ProMeta Version 3.0 (Internovi). Random-effects models were applied throughout, as they account for both within- and between-study variance. ProMeta also generated 95% confidence intervals around the pooled effect size estimates. Heterogeneity was assessed using Cochran’s *Q* and *I*^2^ statistics, with *I*^2^ values of 25%, 50%, and 75% interpreted as low, moderate, and high heterogeneity, respectively.

Potential publication bias was evaluated both visually and statistically. Funnel plots of effect size against standard error were inspected for asymmetry, while Begg and Mazumdar’s rank correlation test and Egger’s regression intercept test provided formal assessments. Finally, all analyses were conducted twice: once including the full set of studies and once excluding potential outliers, defined as studies with statistically significant standardized residuals (*P* < .05).^50^

### Moderator analysis

Across all 3 meta-analyses, we examined whether effect sizes varied as a function of age, country, sex assigned at birth (% of females), BMI, type of HRV measure, ethnicity, health status, and minority type (ethnic vs. sexual). Continuous moderators (sex assigned at birth, age, BMI) were evaluated using meta-regression analysis, whereas categorical moderators (country, ethnicity, health status, minority type) were entered as grouping variables in the effect size calculations. A minimum of 4 studies per subgroup was required for inclusion in the moderation analyses.

## Results

### Differences in HRV between minoritized and majority groups (M1)

**Figure 2:**
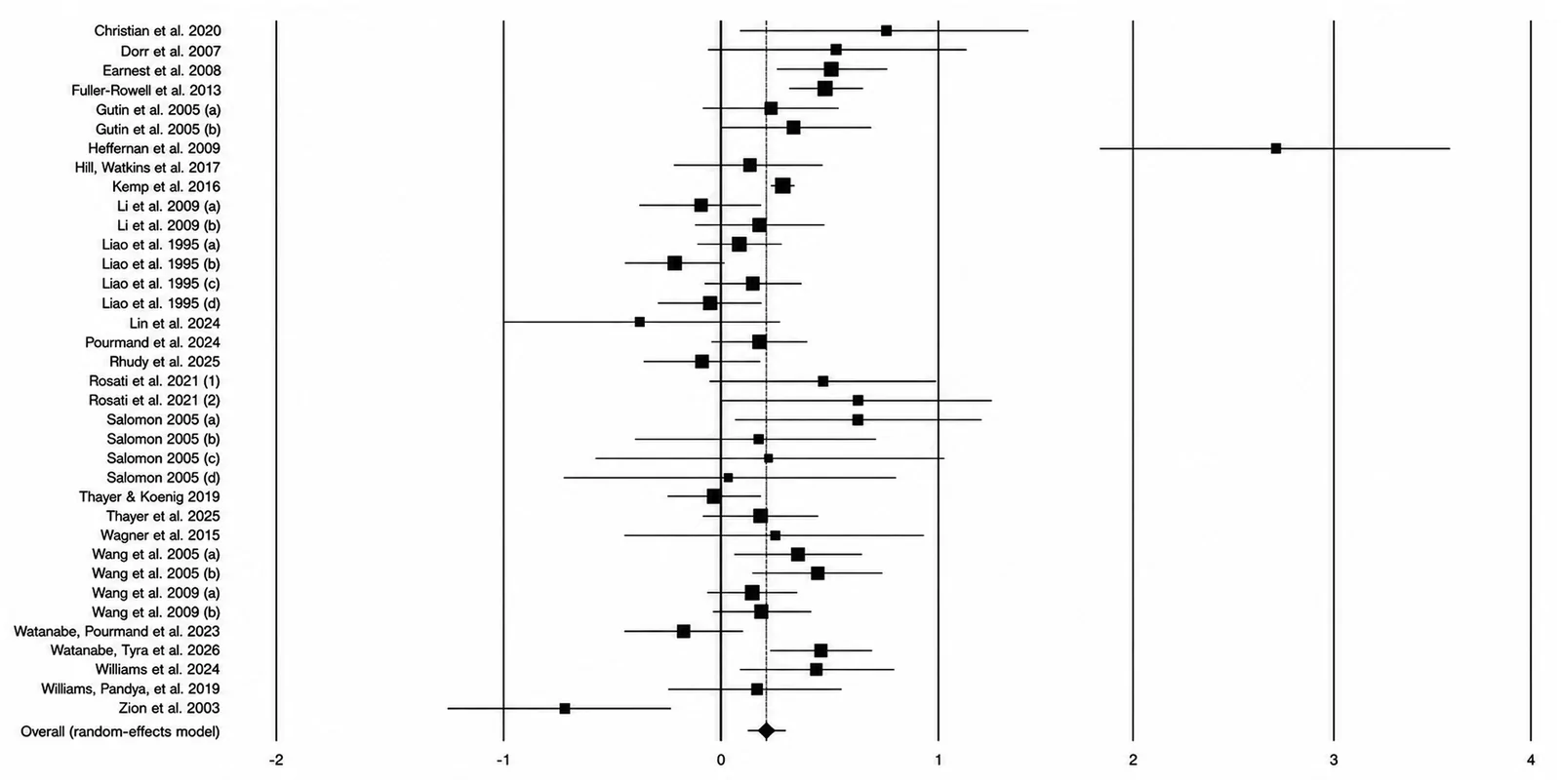
Forest plot for the meta-analysis of studies assessing the difference in HRV between minoritized groups and majority groups (M1). Black squares represent the effect sizes (Hedges’ g) of individual studies, with their corresponding 95% confidence intervals. The black diamond represents the overall pooled effect size and its 95% confidence interval.

Analysis of 36 studies (*n *= 16634; 4551 from minoritized groups and 12083 from majority groups) indicated that HRV was significantly higher in minoritized groups than in majority groups (*g* = .22, 95% CI: 0.13-0.31, *P* < .001, see Figure 2) with substantial heterogeneity across studies (*Q *= 113.56, *P* < .001; *I*^2^ = 73.80). No evidence of publication bias was detected based on Egger’s unweighted regression asymmetry test (intercept = −0.28, *t* = –0.55, *P* = .58) or Begg and Mazumdar’s rank correlation test (*Z *= 1.58; *P* = .11) (see Figure S1a for the funnel plot). Excluding potential outliers^51^^,^^52^ did not alter the overall effect size or heterogeneity (*g* = 0.21, 95% CI: 0.14-0.29, *P* < .001; *Q *= 88.60, *P* < .001; *I*^2^ = 62.75), nor did it affect the tests of publication bias (*Z *= 1.38; *P* = .168; intercept = –0.45, *t* = –1.02, *P* = .31).

Moderation analyses were limited by the available evidence. Due to an insufficient number of studies, it was not possible to evaluate the effects of country of origin (as almost all studies were conducted in the United States), minority type (only one study examined sexual minorities^12^), or type of ethnic minority as most studies focused on AAs except for 2 examining a non-AA minority (Black Brazilian^53^ and Asian American^18^. Meta-regression analyses indicated no significant moderating effects of age (*k *= 34; intercept = 0.25; *P* = .75), sex assigned at birth (*k *= 34; intercept = 0.18; *P* = .70), or BMI (*k *= 18; intercept = −0.84; *P* = .50).

The only significant moderator was the type of HRV measure used (*Q *= 6.07, *P* = .014). Because only 4 studies employed RSA as the HRV metric—a substantially smaller number compared with studies using RMSSD and HF-HRV—we recategorized HRV measures for the moderation analysis to improve statistical stability. Specifically, RMSSD was contrasted with a combined HF-HRV/RSA category, given the closer conceptual overlap between HF-HRV and RSA as respiration-linked indices of vagal cardiac regulation. Larger effects were observed for studies using time-domain measures (*g* = 0.36, *k *= 15, *n *= 3700, *P* < .001) compared to studies using HF-HRV and RSA (*g* = 0.11, *k *= 19, *n *= 12 855, *P* = .079) (Supplementary material S2). Following the exclusion of outliers, this moderation effect became marginally significant (*Q *= 3.53, *P* = .060). Subgroup analyses indicated substantial heterogeneity for RMSSD (*Q *= 61.60, *P* < .001; *I²* = 77.27) and HF-HRV/RSA (*Q *= 65.96, *P* < .001; *I²* = 72.71), which remained significant following the exclusion of outliers.

### The effect of discrimination-related stimuli on HRV (M2)

**Figure 3:**
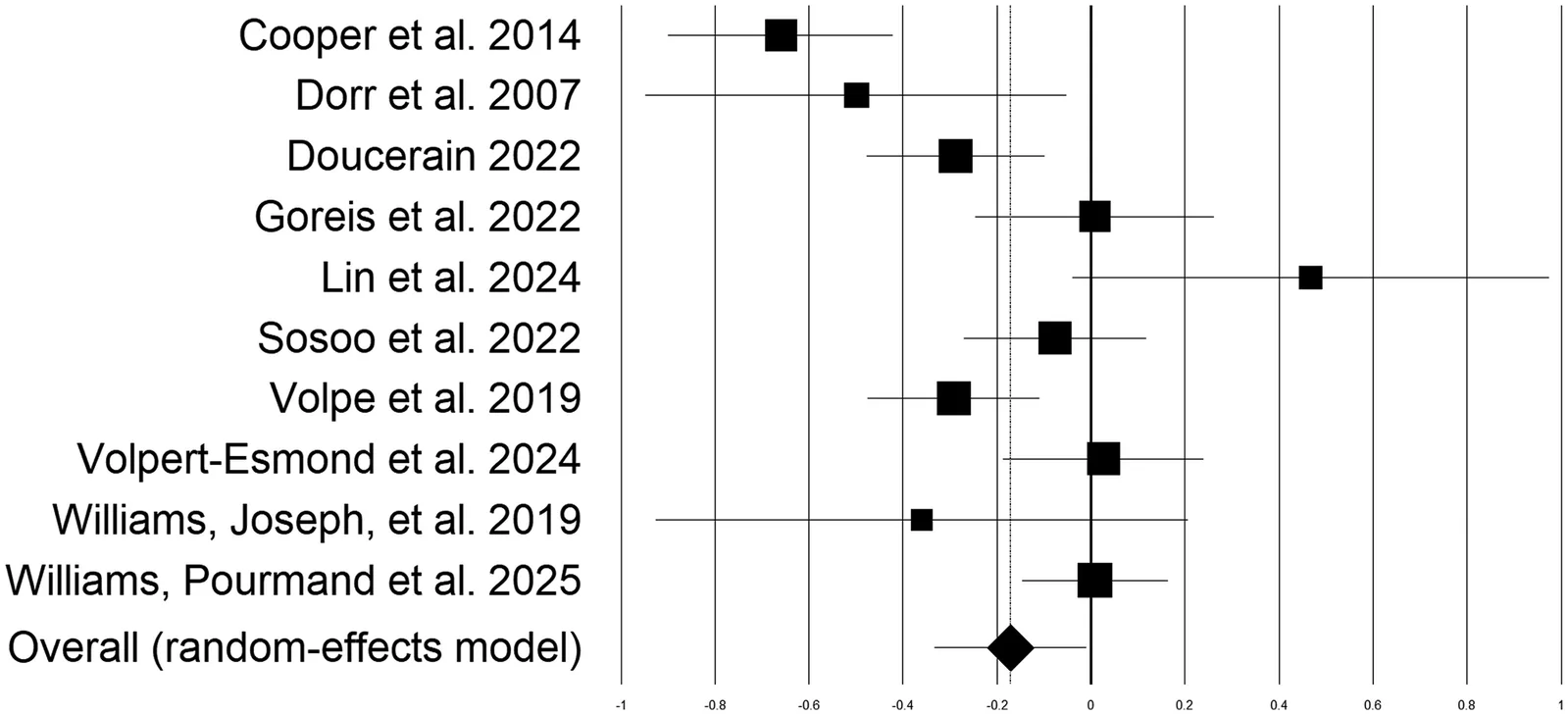
Forest plot for the meta-analysis of studies assessing HRV reactivity to a discrimination-related task (M2). Black squares represent the effect sizes (Hedges’ g) of individual studies, with their corresponding 95% confidence intervals. The black diamond represents the overall pooled effect size and its 95% confidence interval.

The analysis of 10 studies comprising 757 participants revealed a significant decrease in HRV among minoritized individuals during discrimination-related tasks (*g *= −.17, 95% CI: −0.33 to −0.01, *P* = .04, see Figure 3) in a heterogeneous set of studies (*Q *= 39.18, *P* < .001; *I*^2^ = 77.03). We found no evidence of publication bias on the classic funnel plot (Supplementary material S1b), Begg’s rank test (*Z* = 0.27; *P* = .79), or Egger’s regression test (intercept = −0.41, *t *= −0.21, *P* = .84). Excluding potential outliers^18^^,^^54^ did not substantially alter the overall effect size or heterogeneity (*g *= −0.14, 95% CI: −0.26 to −0.02, *P* = .03; *Q *= 15.55, *P* = .03; *I*^2^ = 54.98) or the tests of publication bias (*Z* = 0.00; *P *= 1.00; intercept = −1.56, *t *= −0.97, *P* = .37).

Due to an insufficient number of studies, it was not possible to evaluate the effects of country of origin, as only 2 studies were conducted outside the United States.^39^^,^^40^ Similarly, all included studies investigated ethnic minority populations, with 7 focusing on AA participants and 3 examining a non-AA minority group (Turkish minority^40^ and Latin/Hispanic).^42^^,^^55^

No moderation effects emerged from the exploratory meta-regression analyses conducted on age (*k *= 9; intercept = −0.07; *P* = .90), sex assigned at birth (*k *= 8; intercept = −0.06; *P* = .83), or BMI (*k *= 5; intercept = −1.32; *P* = .80).

### Association between HRV and self-reported perceived discrimination (M3)

**Figure 4:**
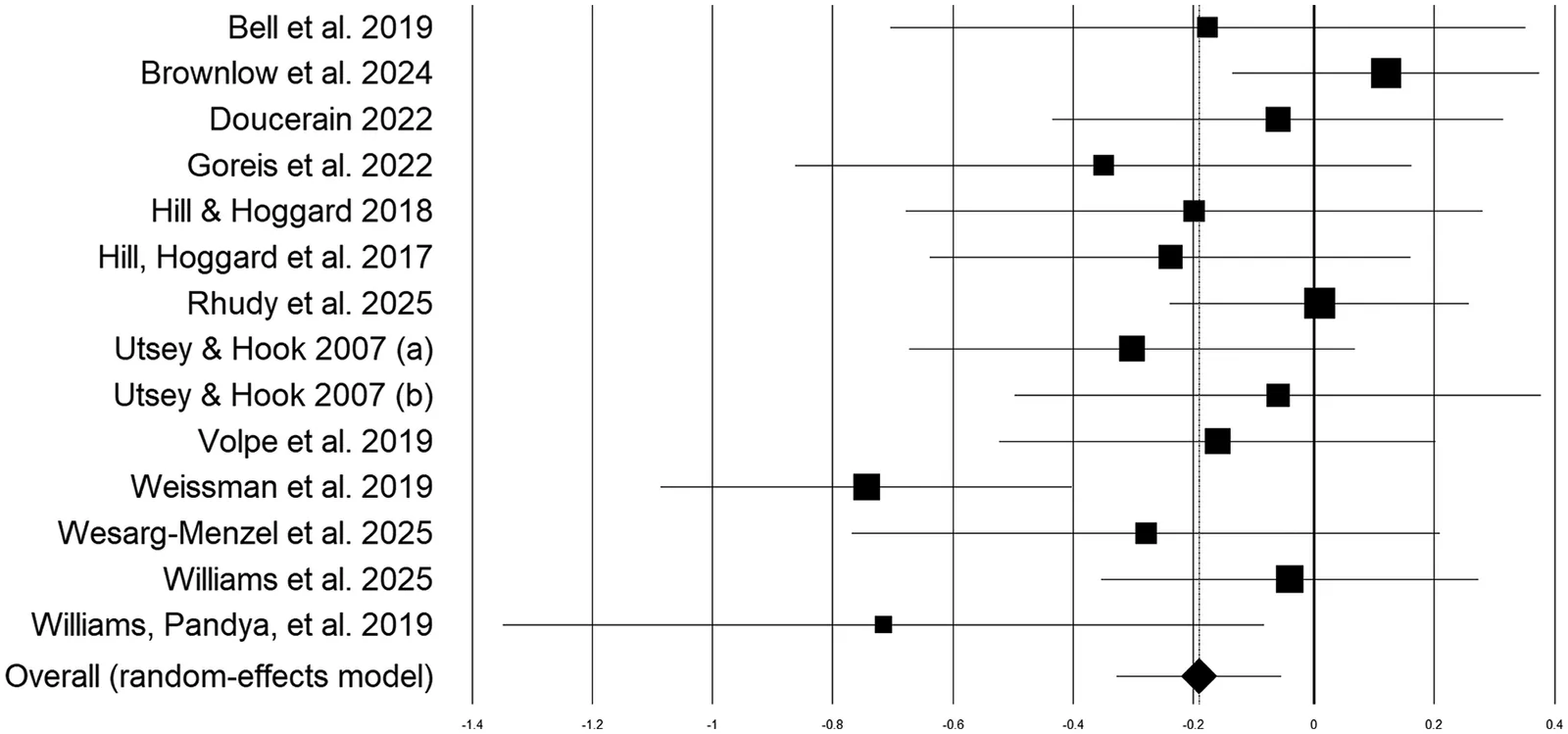
Forest plot for the meta-analysis of studies assessing the association between tonic HRV and self-reported perceived discrimination (M3). Black squares represent the effect sizes (Hedges’ g) of individual studies, with their corresponding 95% confidence intervals. The black diamond represents the overall pooled effect size and its 95% confidence interval..

The analysis of 14 studies including 1620 participants revealed a significant negative association between HRV and perceived discrimination (*g *= −.19, 95% CI: −0.33 to −0.05, *P* = .006; see Figure 4). Significant heterogeneity was observed across studies (*Q *= 23.07, *P* = .04; *I*^2^ = 43.64). Evidence of publication bias was indicated by Egger’s regression (intercept = –2.67, *t *= −2.22, *P* = .05) and Begg’s rank correlation test (*Z *= −2.14; *P* = .03) (Supplementary material S1c).

Exclusion of one potential outlier^45^ attenuated but did not eliminate the association (*g *= −0.10, 95% CI: −0.21 to −0.00, *P* = .047), while evidence of publication bias remained based on Begg’s test (*Z *= −2.68; *P* = .007) and Egger’s test (intercept = −2.85, *t* = –5.15, *P* < .001), and heterogeneity was substantially reduced (*Q *= 10.81, *P* = .545; *I*^2^ = 0.00).

Moderator analysis was limited by the available evidence. The moderating effect of country of origin could not be evaluated, as only 3 studies were conducted outside the United States.^39^^,^^40^^,^^56^ Likewise, all included studies examined ethnic minorities, with 6 studies focusing on non-African American groups (Turkish minority in Goreis et al.^40^; Mexican minority in Weissman et al.^45^; Arabic speaking refugees in Wesarg-Menzel et al.^56^; Latin in Williams et al.^42^; Native American in Rhudy et al.^38^; and North African in Doucerain^39^). Exploratory meta-regression analyses identified no significant moderating effects for age (*k *= 12; intercept = –0.77; *P* = .17), sex assigned at birth (*k *= 12; intercept = –0.16; *P* = .98), or BMI (*k *= 7; intercept = –1.85; *P* = .32).

## Discussion

This meta-analytic review offers an integrated account of how discrimination is associated with cardiac vagal regulation in minoritized populations, drawing together 3 complementary findings: minoritized groups exhibit higher resting HRV than majority groups (M1); discrimination-related stimuli elicit acute reductions in HRV (M2); and greater perceived discrimination is associated with lower resting HRV (M3).

Consistent with previous studies on the “Cardiovascular Conundrum,”^9^ minoritized individuals—predominantly AAs—showed significantly higher resting HRV than their majority counterparts. In healthy populations, higher HRV is typically interpreted as a marker of flexible parasympathetic control and stress resilience.^57^ Yet, minoritized groups bear disproportionate cardiovascular risk, including elevated blood pressure and greater TPR, resulting in the paradoxical co-occurrence of elevated HRV and an adverse hemodynamic profile.^10^ One plausible interpretation is compensatory baroreflex engagement or sustained regulatory effort, shaped by chronic coping demands associated with discrimination. Although this interpretation is consistent with previous theoretical and empirical work, the present meta-analysis did not directly assess baroreflex function, vascular resistance, or neural mechanisms. Therefore, these processes should be considered putative mechanisms rather than conclusions that can be drawn directly from the current findings.

Successful emotion regulation is associated with both greater resting HRV as well as HRV increases during emotion regulation including reappraisal and suppression.^17^ Indeed, greater HF-HRV has been associated with increased activity in prefrontal brain regions involved in cognitive control and emotion regulation, coupled with reduced subcortical activity in regions such as the amygdala.^58^ In parallel, behavioral evidence indicates that AAs often inhibit or suppress anger in response to unfair treatment.^20^^,^^59^ Limited evidence further suggests that sexual minoritized samples may exhibit a similar HRV-TPR dynamic,^12^ reinforcing the hypothesis that minority stress, rather than genetics per se, may contribute to this paradoxical vagal-vascular profile. African American and sexual minority groups share a longstanding history of exposure to unfair treatment and internalized stigma, in contexts in which formal civil rights protections have been established relatively recently.

Our second meta-analysis (M2) demonstrated that discrimina­tion-related stimuli reliably elicited reductions in ­phasic HRV among minoritized individuals, consistent with the prototypical fight-or-flight response. Although these decreases were modest in magnitude compared to predictions derived from traditional stress reactivity models, current findings suggest that—even in groups with elevated baseline HRV—exposure to discrimination cues is associated with transient vagal withdrawal. This pattern aligns with evidence that engaging in discrimination-related tasks, such as recalling or discussing personal experiences of discrimination, is physiologically stressful, consistent with a threat response. Such changes mirror the autonomic adjustments typically observed in response to psychosocial challenge.^60^ For instance, Dorr et al.^20^ reported that AA participants, relative to EAs, exhibited heightened cardiac reactivity—indexed by systolic blood pressure, cardiac output, and heart rate—together with greater decreases in HRV when debating racially charged topics. Similarly, Armstead et al.^61^ observed greater blood pressure reactivity among AA women exposed to racist provocations compared to non-racist stimuli, while McNeilly et al.^62^ found stronger blood pressure responses to race-themed debates relative to neutral debates in AA women. In line with these findings, Cooper et al.^54^ showed that exposure to discriminatory contexts evoked significant vagal withdrawal alongside vascular activation, underscoring the role of acute discrimination as a potent autonomic stressor. Taken together, these results suggest that discrimination-related stress may be associated with physiolo­gical costs linked to increased vulnerability for a variety of conditions.

Notably, higher levels of self-reported perceived discrimination were associated with lower resting HRV (M3). In AAs, greater perceived discrimination has been specifically associated with reduced HF-HRV,^42^ with evidence that these effects are mediated by rumination and anger.^63^ Rumination has been linked to lower HRV as well as heightened vascular responses, including elevated TPR and blood pressure.^16^^,^^64^ Such vagal reductions may extend into nocturnal periods, where lower HRV was linked to feelings of sadness, hopelessness, frustration, and powerlessness in response to racism. These findings converge with broader research on negative affectivity, where neuroticism and rumination predict persistent reductions in HRV across both wake and sleep states.^14^ Importantly, rumination about racist encounters may sustain autonomic activation beyond the acute event and has been linked to alterations in physiological functioning.^65^ Such processes may represent one plausible psychophysiological pathway linking chronic discrimination with cardiovascular disease risk and immune dysregulation.^66^ Trait rumination—a cognitive style strongly tied to depression and anxiety—has similarly been shown to influence both blood pressure and vagally mediated HRV and may moderate the autonomic consequences of chronic exposure to racism.^16^^,^^67^ This interpretation is consistent with the Biopsychosocial Model of Racism,^4^ which conceptualizes rumination and emotional suppression as prototypical responses to race-related stress. Notably, race-related vigilance has been associated with depressive symptoms via rumination among AAs.^67–70^

However, the association between low perceived discrimination and high resting HRV may reflect a core feature of the cardiovascular conundrum, particularly in contexts where discriminatory experiences are less consciously appraised or become routinized over time. One possible interpretation is that individuals repeatedly exposed to discriminatory environments may increasingly rely on the habitual suppression of negative emotional responses as a coping strategy. Such sustained regulatory effort may be reflected in higher resting HRV, consistent with increased engagement of top–down emotion regulatory processes.^17^ However, over time, these responses may become automatized and less accessible to conscious awareness while potentially being associated with physiological costs, potentially including elevated TPR, a well-established risk factor for cardiovascular morbidity. Within ethnically minoritized groups, this process may be further shaped by internalized forms of stigma, which have been linked to adverse psychological and somatic outcomes.^71^ It is important to note, however, that evidence of publication bias was detected for this meta-analysis. Although the direction of the association appears to be consistent across studies, its magnitude may be inflated due to the selective publication of statistically significant findings. Therefore, these findings should be interpreted with caution.

Overall, these findings are consistent with the possibility of distinct detrimental pathways in minoritized groups, in line with evidence showing that both anger expression and anger inhibition among Black individuals are associated with heightened vascular reactivity, an effect that may contribute to their elevated risk for hypertension-related morbidity and mortality.^20^

Importantly, the present meta-analysis does not allow a definitive adjudication of the functional meaning of elevated tonic HRV in minoritized populations. Rather, the observed pattern is most consistent with a context-dependent interpretation of HRV, in which elevated parasympathetic indices may reflect both adaptive regulatory engagement and the physiological signature of chronic coping demands. Disentangling these alternatives will require longitudinal and experimental designs capable of tracking within-person changes in HRV alongside concurrent measures of vascular functioning and emotion regulation processes.

Unfortunately, moderator analyses were constrained by the limited coverage of the available literature. Our analyses showed that group differences were more robust for time-domain compared with frequency-domain measures of HRV. One possible explanation is that RMSSD, the most commonly used time-domain index, presents several methodological advantages over HF-HRV and RSA, particularly in discrimination research contexts. Specifically, RMSSD is less influenced by respiratory patterns and artifact contamination and is generally considered more reliable for short-term recordings, which characterize many studies assessing discrimination-related stress.^72^ However, these methodological considerations should be regarded as plausible hypotheses rather than definitive explanations for the observed differences across HRV measures. Direct comparisons of multiple vagally-mediated HRV indices within the same study will be important to test these hypotheses. Accordingly, the present moderation finding highlights the value of multimethod HRV assessment, suggesting that future studies should systematically include multiple vagally-mediated HRV indices to determine whether elevated resting HRV in minoritized groups reflects a generalized phenomenon or is partly dependent on the specific HRV metric used.

Although age, sex assigned at birth, and BMI did not emerge as significant moderators across the present meta-analyses, these findings should be interpreted with considerable caution given the limited number of available studies and the resulting low statistical power of these exploratory moderation analyses. Consequently, the absence of statistically significant moderation should not be interpreted as evidence that these variables do not influence the association between discrimination and parasympathetic regulation. Rather, the present null findings should be regarded as preliminary rather than conclusive.

Based on prior literature, age and sex assigned at birth remain plausible candidate moderators of the association between discrimination and parasympathetic regulation. Women generally exhibit higher resting parasympathetic tone,^32^ but also appear more physiologically responsive to psychosocial stressors, often showing larger HRV reductions during emotionally salient contexts.^34^ Similarly, developmental stage may be particularly relevant, as adolescence and young adulthood are characterized by ongoing identity formation, heightened sensitivity to social evaluation, and increased exposure to discriminatory experiences, all of which may amplify autonomic responses to stress.^73^ From a life-course perspective, repeated exposure to discrimination during sensitive developmental periods may contribute to the biological embedding of stress and may be associated with progressive changes in autonomic and cardiovascular functioning across adulthood.^74^ However, the current evidence base remains too limited and unbalanced to adequately evaluate these potential moderating effects. Future studies should prioritize recruiting participants across a broader age range and ensure adequate representation of both sexes in order to clarify the potential moderating role of developmental and sex-related factors in the relationship between discrimination and vagally mediated HRV.

### Limitations and future directions

Several limitations of the present meta-analyses should be acknowledged. First, only studies published in the English language were included, which may have led to language bias and limited the scope of available evidence. Second, the exclusion of gray literature may have introduced publication bias, as unpublished or non–peer-reviewed studies are less likely to report null or smaller effects, potentially leading to an overestimation of the observed effect sizes. Third, the substantial heterogeneity observed across analyses further suggests that the relationship between discrimination and HRV is unlikely to be uniform across contexts and populations. Variability in ­sociocultural context, developmental stage, health status, minority identity, discrimination exposure, and HRV assessment procedures may all shape the magnitude and direction of these associations. Accordingly, the pooled effects identified in the present meta-analysis should be interpreted as broad trends rather than universal psychophysiological responses.

A further conceptual limitation concerns the extent to which the present findings can be generalized beyond the populations directly represented in the included studies. Although the theoretical framework of minority stress suggests that similar psychophysiological mechanisms may extend to other socially marginalized groups, such as sexual minorities, the empirical evidence included in this meta-analysis is predominantly based on African American participants. At present, it remains unclear whether the cardiovascular conundrum generalizes to other ethnic groups, as preliminary findings suggest that Asian and Hispanic populations may not exhibit the same physiological pattern.^75^

As a result, any extrapolation of the observed HRV patterns to other minoritized populations should be considered speculative. Future research should therefore explicitly compare across multiple minoritized groups within the same methodological framework in order to determine whether the observed patterns reflect a general mechanism of social stress exposure or are instead ­specific to particular forms of marginalization.

Moreover, the number of eligible studies was modest in some analyses (particularly M2 and M3), reducing the statistical power for detecting moderator effects. Finally, the predominance of cross-sectional designs limits causal inference about the temporal sequence linking discrimination encounters, coping strategies, and parasympathetic regulation. It is also possible that higher HRV may buffer individuals from perceiving discrimination^76^; however, its effects may nonetheless manifest at the physiological level, for instance through increased TPR.^77^

Future research should move beyond correlational approaches and adopt experimental and longitudinal designs that more directly build on the patterns identified in the present meta-analysis. First, in light of the finding that minoritized groups show higher resting (tonic) HRV (M1), future studies should directly test whether this pattern reflects adaptive regulatory capacity versus sustained compensatory regulation under chronic stress. Experimental paradigms manipulating coping strategies (eg, anger suppression vs. cognitive reappraisal) in the context of minority stress-related versus neutral stressors would allow for a more precise examination of how regulatory effort relates to cardiovascular outcomes, including HRV, total peripheral resistance, and blood pressure.

Second, consistent with the observed acute reductions in phasic HRV during discrimination-related stimuli (M2), laboratory studies should further disentangle the temporal dynamics of autonomic responding by examining whether individuals with elevated resting HRV show attenuated or exaggerated vagal withdrawal under standardized discrimination paradigms, and whether these responses habituate or sensitize over repeated exposures.

Third, in line with the association between greater perceived discrimination and lower resting HRV (M3), future longitudinal and ambulatory studies are needed to clarify how cumulative exposure to discrimination relates to longer-term trajectories of autonomic regulation. Ecological momentary assessment combined with ambulatory monitoring of heart rate, blood pressure, and HRV would be particularly valuable for capturing how daily discrimination experiences and coping processes translate into physiological change in real-world contexts.

Finally, longitudinal and multimodal approaches integrating HRV with complementary indices such as baroreflex sensitivity and neural markers of emotion regulation should be prioritized. Such designs would help clarify whether elevated HRV in minoritized groups reflects adaptive vagal engagement, maladaptive compensatory regulation, or a dynamic combination of both across time, and would further elucidate the pathways linking acute stress reactivity to chronic parasympathetic–vascular dysregulation.

Overall, by explicitly building on the distinct patterns observed across tonic HRV differences (M1), phasic reactivity (M2), and perceived discrimination associations (M3), future research can more precisely identify the mechanisms underlying the cardiovascular conundrum and inform potential targets for intervention aimed at preserving autonomic flexibility and reducing long-term cardiovascular risk.

## Supplementary Material

kaag058_Supplementary_Data

## Data Availability

This study did not produce new data.

## Study registration

This study was not formally registered.

## Analytic plan pre-registration

The analysis plan was not formally pre-registered.

## Analytic code availability

There is no analytic code associated with this study.

## Materials availability

ProMeta files used to conduct the meta-analyses may be available by emailing the corresponding author.
